# Supplementary material for: Stacking Model‐Based Classifiers for Dealing With Multiple Sets of Noisy Labels
Source: Biom J. 2025 Mar 12;67(2):e70042. doi: 10.1002/bimj.70042 (PMC11898607; doi:10.1002/bimj.70042)
Supplement: Supplementary file 1 — Supporting Information [file BIMJ-67-e70042-s001.zip › density-based-ensemble-model-main/competitors_real_data/netlab-master/netlab-master/help/demhmc1.htm]

Netlab Reference Manual demhmc1


# demhmc1

## Purpose

Demonstrate Hybrid Monte Carlo sampling on mixture of two Gaussians.

## Synopsis

```
demhmc1
```

## Description

The problem consists of generating data from a mixture of two Gaussians
in two dimensions using a hybrid Monte Carlo algorithm with persistence.
A mixture model is then fitted to the sample to compare it with the
true underlying generator.

## See Also

`demhmc3`, `hmc`, `dempot`, `demgpot`

---

**Pages:**
Index


---

Copyright (c) Ian T Nabney (1996-9)
